# Attention‐guided duplex adversarial U‐net for pancreatic segmentation from computed tomography images

**DOI:** 10.1002/acm2.13537

**Published:** 2022-02-24

**Authors:** Meiyu Li, Fenghui Lian, Yang Li, Shuxu Guo

**Affiliations:** ^1^ College of Electronic Science and Engineering Jilin University Changchun China; ^2^ School of Aviation Operations and Services Air Force Aviation University Changchun China

**Keywords:** attention block, backbone segmentor, generative adversarial network, pancreatic segmentation

## Abstract

**Purpose:**

Segmenting the organs from computed tomography (CT) images is crucial to early diagnosis and treatment. Pancreas segmentation is especially challenging because the pancreas has a small volume and a large variation in shape.

**Methods:**

To mitigate this issue, an attention‐guided duplex adversarial U‐Net (ADAU‐Net) for pancreas segmentation is proposed in this work. First, two adversarial networks are integrated into the baseline U‐Net to ensure the obtained prediction maps resemble the ground truths. Then, attention blocks are applied to preserve much contextual information for segmentation. The implementation of the proposed ADAU‐Net consists of two steps: 1) backbone segmentor selection scheme is introduced to select an optimal backbone segmentor from three two‐dimensional segmentation model variants based on a conventional U‐Net and 2) attention blocks are integrated into the backbone segmentor at several locations to enhance the interdependency among pixels for a better segmentation performance, and the optimal structure is selected as a final version.

**Results:**

The experimental results on the National Institutes of Health Pancreas‐CT dataset show that our proposed ADAU‐Net outperforms the baseline segmentation network by 6.39% in dice similarity coefficient and obtains a competitive performance compared with the‐state‐of‐art methods for pancreas segmentation.

**Conclusion:**

The ADAU‐Net achieves satisfactory segmentation results on the public pancreas dataset, indicating that the proposed model can segment pancreas outlines from CT images accurately.

## 1 INTRODUCTION

Computed tomography (CT) is one of the most recognized scanning technologies that are extensively applied to the diagnosis of clinical diseases. Precise CT images analysis is significant for doctors to perform suitable and timely treatments according to the position, shape, and lesion of the abdominal organs. Accurate organs segmentation is a prerequisite for quantitative and qualitative CT scans analysis, and it is urgent to investigate automatic organ segmentation methods. With the development of deep learning technology in recent years, convolutional neural networks (CNNs) have been widely used in medical imaging processing. Several CNN‐based organ segmentation methods have made great achievements.^1^, ^2^, ^3^, ^4^ Chlebus et al.^1^ introduced an object‐based postprocessing step into a two‐dimensional (2D) fully convolutional neural network (FCN) for automatic liver tumor segmentation. Cascaded with a model that was trained with object‐level hand‐crafted features, the 2D FCN reduced the false‐positive findings and improved the tumors segmentation quality. Tong et al.^2^ developed a fully automated segmentation method for segmenting head and neck organs at risk from CT and low‐field magnetic resonant images, where a fully convolutional DenseNet was used as a segmentation network for voxel‐wise prediction and a CNN was involved as a discriminator network for correcting prediction errors and image‐level inconsistency. Compared with bulky organs, such as the liver, spleen, kidneys, and bladder, pancreas segmentation is more challenging. This is mainly because the pancreas accounts for a limited proportion in the abdominal CT volume, and the pancreas of different individuals varies significantly in size, shape, and location. Meanwhile, other adjacent abdominal organs often affect the appearance properties of the pancreas, and the degree of boundary contrast is influenced by the surrounding visceral fat tissues. Besides, the image motion artifacts, along with some other factors, may also affect the texture of the pancreas. All these aspects make pancreas segmentation a challenging task.

Some deep learning‐based models have been proposed for pancreas segmentation.^5^, ^6^, ^7^ Roth et al.^5^ firstly exploited holistically‐nested convolutional networks to localize the pancreas from three‐dimensional (3D) computed tomography. Then, mid‐level cues were collected from the organ interior and boundary maps to achieve accurate pancreas localization and segmentation. Cai et al.^6^ proposed a CNN based on bi‐directional convolutional long short‐term memory to weaken the spatial non‐smoothness among contiguous slices. Also, they presented an effective architecture for pancreas segmentation. These research works show that the methods based on deep learning are effective for pancreas segmentation. However, the 3D methods always increase time cost and have a high requirement for server configuration, while the simple 2D models usually ignore the spatial details from adjacent slices. Thus, the existing models for pancreas segmentation can be further improved.

Attention mechanism^8^ was initially proposed in the field of natural language processing, and nowadays it has been extensively used with CNNs for various tasks.^9^, ^10^, ^11^, ^12^ Oktay et al.^9^ presented an attention gate model that can automatically focus on target structures for medical imaging. This proposed attention gate can be integrated into standard CNN architectures to achieve high sensitivity and prediction accuracy through highlighting useful regions while suppressing irrelevant regions. Liu et al.^10^ introduced an attention module into a CNN for accurate segmentation and quantification of ischemic stroke and white matter hyperintensities lesions. Based on this, the target areas can be effectively distinguished from the background. These research works indicate that integrating attention mechanisms with neural networks is a promising approach for organ segmentation.

A generative adversarial network (GAN)^13^ is a special CNN consisting of a discriminator and a generator in a conventional version, where these two parts compete with each other in a min‐max two‐player game. GAN has been involved in several organ segmentation tasks for its good performance in several imaging processing fields.^14^, ^15^, ^16^ This paper proposes an attention‐guided dual adversarial U‐Net for pancreas segmentation, called ADAU‐Net. Dual adversarial networks and attention guidance are integrated into a conventional 2D segmentation model to obtain a better segmentor. To our best knowledge, this is the first dual adversarial network with an attention mechanism for pancreas segmentation.

## 2 MATERIALS AND METHODS

### 2.1 Dataset and evaluation metrics

The National Institutes of Health (NIH) pancreas segmentation dataset^17^, ^18^ contains 82 contrast‐enhanced abdominal CT volumes, and it is the most recognized public dataset for pancreas segmentation. Each CT scan has a resolution of 512×512×L, and L varies from patient to patient within the range of 181–466. In this work, the CT scans are resized to [208, 208] based on the approximate range of the pancreas label in the scans to ensure that each slice contains complete pancreas areas. Meanwhile, the CT volumes are randomly split into four folds, where three folds are used for training and the remaining one is used for testing, that is, 4‐fold cross‐validation. Metrics dice similarity coefficient (DSC) and Jaccard are used to evaluate the similarity between the obtained prediction maps and their corresponding ground truths. Besides, average symmetric surface distance (ASD) and root‐mean‐squared error (RMSE) are used to determine whether the pancreas edge is well segmented compared with the edge of the ground truths. Our algorithm is implemented by PyTorch environment,^19^ and the ADAU‐Net processing is conducted on one NVIDIA GeForce GTX 1080Ti GPU with 11 GB memory. In the experiment, Adam optimizer is used with the learning rate of 0.0001, and the momentum of 0.9 and 0.99. The networks are optimized from scratch with a batch size of 1.

### 2.2 Generative adversarial networks

GAN^13^ is an emerging deep learning model that consists of a generator G and a discriminator D. G produces fake samples from random noise to fool D, while D attempts to distinguish between the produced samples and real images.^13^ In the training procedure, G and D update synchronously until reaching Nash equilibrium, and at this time, G and D both achieve optimal performance.

### 2.3 Attention mechanism

Generally, attention mechanism^8^ guides to allocate existing processing resources in favor of the most informative parts of the input, and it is analogous to the observation system of humans. When we observe external things, we tend to first selectively obtain the important parts according to our needs. Then, we combine the information of different regions to form an overall impression of the observed things. Similarly, the attention mechanism generates a context vector that assigns weights to the input sequence to localize the informative regions.^20^ In this way, it focuses on the significant features while the remaining parts of the inputs receive relatively little attention. Based on this, the interdependency among pixels is enhanced, and rich contextual information is captured, contributing to a more contextualized prediction.^8^

### 2.4 The proposed segmentor ADAU‐Net

**FIGURE 1:**
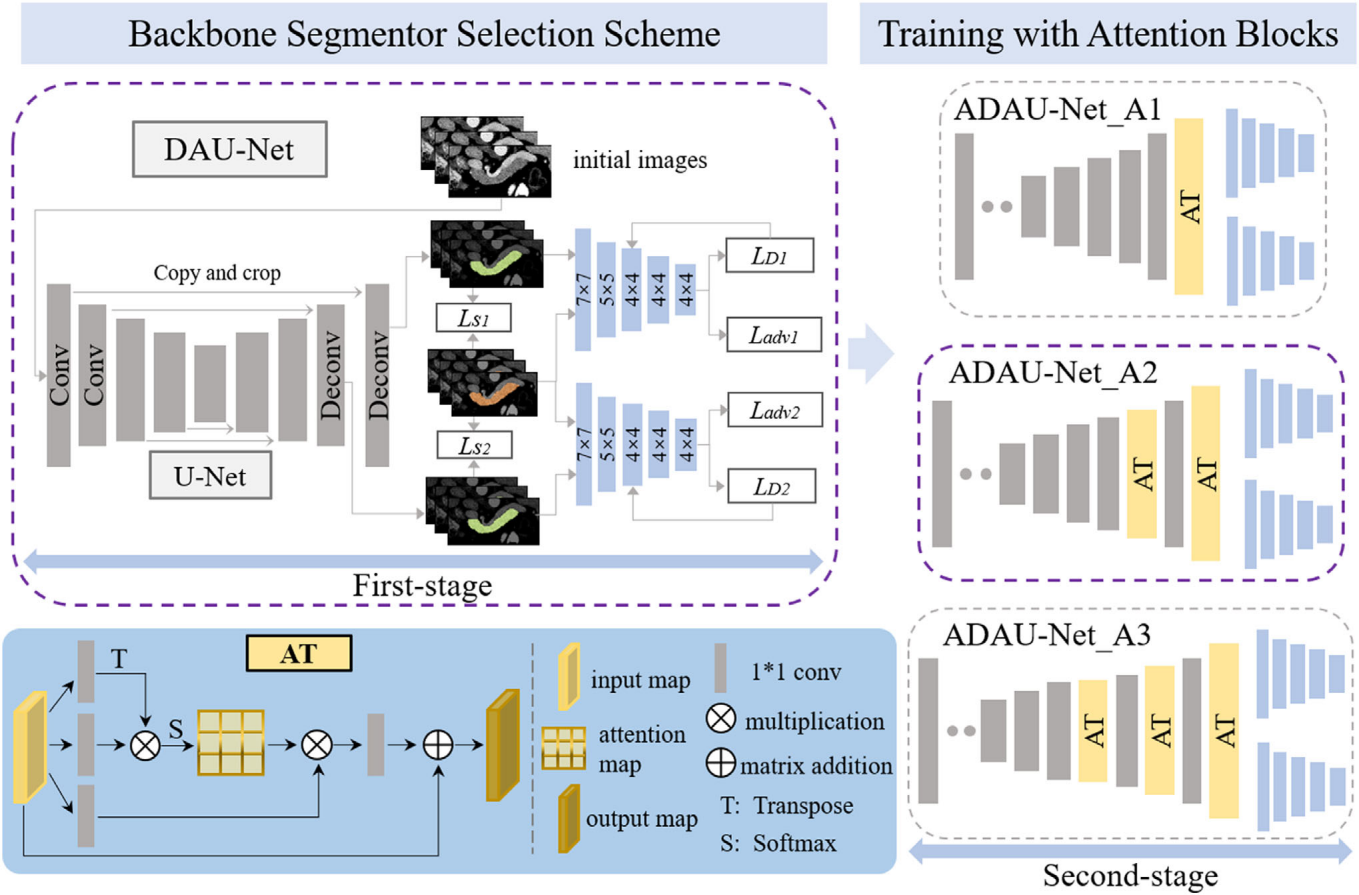
Schematic of the proposed attention‐guided duplex adversarial U‐Net (ADAU‐Net), which mainly contains two steps: backbone segmentor selection scheme and training with attention blocks module. The gray boxes in the first and second‐stage represent the convolutional layers in the segmentation model, the blue boxes in the first and second stages represent the convolutional layers in discriminator, and the yellow boxes AT in second‐stage represent the attention blocks involved in this work

In this section, the proposed model ADAU‐Net is described. To achieve a better performance, the ADAU‐Net executes in two steps: backbone segmentor selection and training with attention blocks. First, a backbone segmentor selection scheme is designed to find an optimal segmentation network as the backbone framework in our proposed algorithm. Second, the selected backbone segmentor is trained with attention blocks to further boost the segmentation performance. The detailed implementation of the ADAU‐Net is shown in Figure 1.

#### 2.4.1 Backbone segmentor selection scheme

As for backbone segmentor selection, three groups of segmentation models are set, and the one with optimal performance is selected as the backbone of our proposed ADAU‐Net. Specifically, the U‐Net proposed by Ronneberger et al.^21^ is first adopted as the basic segmentation model. Then, an adversarial network is introduced into the U‐Net to improve its segmentation performance, and the network is called AU‐Net. There are five convolutional kernels in the discriminator of the AU‐Net, and the corresponding sizes are 4×4, 4×4, 4×4, 5×5, and 7×7. Next, a segmentor called DAU‐Net is proposed, and it imposes an extra constraint on the AU‐Net by integrating adversarial learning into the updated U‐Net model.

**FIGURE 2:**
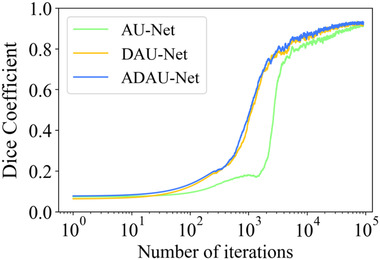
Dice similarity coefficient (DSC) curves of adversarial U‐Net (AU‐Net), duplex adversarial U‐Net (DAU‐Net), and attention‐guided duplex adversarial U‐Net (ADAU‐Net) in the training process

In DAU‐Net, dual adversarial networks are integrated into the baseline U‐Net to obtain a better segmentor. The involvement of a GAN helps to make the predicted probability maps from the baseline U‐Net much similar to the ground truths, as GAN can model data distributions through its special competing mechanism. Considering the equipment in this study, GAN is introduced into this existing adversarial U‐Net once more to further ensure the obtained prediction maps resemble the ground truths to search for a better model for pancreas segmentation. Especially, the double extra constraints from the dual adversarial networks can improve the network performance of the AU‐Net to some extent. As can be seen from Figure 2, compared to AU‐Net, the DAU‐Net improves the convergence speed and obtains a better segmentation performance in the training process. The discriminators used in the DAU‐Net have the same structures as the adversarial network in the AU‐Net.

The energy function of the baseline U‐Net is defined as Equation (1), where $I^{S}$ refers to the obtained maps from the segmentation network while $I^{T}$ refers to the corresponding ground truths.

(1) $$1-\frac{2\left|I^{S}\cap I^{T}\right|}{\left|I^{S}\right|+\left|I^{T}\right|}$$

As shown in Equations (2) and (3), the model AU‐Net includes a segmentation loss for the segmentor and an adversarial loss for the discriminator. Equation (2) consists of two parts, where the former represents the loss function of a conventional U‐Net, and the latter represents extra guidance from the adversarial network. (*fake*) and (*truth*) respectively denote the distribution of the synthetic samples and the original dataset. θ_D_ and θ_G_ respectively refer to the parameters in the discriminator and the generator. *D*_θD_ (*G*_θG_ (*I^S^*)) represents the probability that the input of D comes from the synthetic samples, while *D*_θD_ (*I^T^*) represents the probability that the input of D comes from the original dataset.

(2) $$1-\frac{2\left|I^{S}\cap I^{T}\right|}{\left|I^{S}\right|+\left|I^{T}\right|}+E_{I^{S}-P_{G}\left(fake\right)}\log\left(1-D_{\theta_{D}}\left(G_{\theta_{G}}\left(I^{S}\right)\right)\right)$$

(3) $$\begin{array}{ccc} & & -E_{I^{T}∼P_{D}\left(truth\right)}\log\left(D_{\theta_{D}}\left(I^{T}\right)\right)\\ & & -E_{I^{S}∼P_{G}\left(fake\right)}\log\left(1-D_{\theta_{D}}\left(G_{\theta_{G}}\left(I^{S}\right)\right)\right) \end{array}$$

The energy function of the segmentation network in the DAU‐Net is defined in Equation (4). It consists of four items: the first and the third items (L_S1_ and L_S2_ in Figure 1) represent the loss functions from the conventional U‐Net, and they are the main components; the second and the fourth items (L_adv1_ and L_adv2_ in Figure 1) represent the extra guidance from the adversarial networks, and they are auxiliary components. $I_{1}^{S}$ and $I_{2}^{S}$ respectively refer to the maps obtained from the last and the penultimate deconvolutional layers in the segmentation network, while $I_{1}^{T}$ and $I_{2}^{T}$ refer to the corresponding ground truths. According to the master‐subordinate relationship, β, γ, ε, and μ are set to 1, 0.004, 0.1, and 0.0004 empirically.

(4) $$\begin{array}{ccc} \beta & \times & \left[1-\frac{2\left|I_{1}^{S}\cap I_{1}^{T}\right|}{\left|I_{1}^{S}\right|+\left|I_{1}^{T}\right|}\right]+\gamma \times E_{I_{1}^{S}∼P_{G1}\left(fake\right)}\\ & & \times \;\log\left(1-D_{\theta_{D1}}\left(G_{\theta_{G1}}\left(I_{1}^{S}\right)\right)\right)\\ & & +\;\varepsilon \times \left[1-\frac{2\left|I_{2}^{S}\cap I_{2}^{T}\right|}{\left|I_{2}^{S}\right|+\left|I_{2}^{T}\right|}\right]+\mu \times E_{I_{2}^{S}∼P_{G2}\left(fake\right)}\\ & & \times \;\log\left(1-D_{\theta_{D2}}\left(G_{\theta_{G2}}\left(I_{2}^{S}\right)\right)\right)\; \end{array}$$

The loss functions of the discriminators of the involved segmentation networks mentioned above are defined in Equations (5) and (6), and they are denoted as L_D1_ and L_D2_ in Figure 1.

(5) $$\begin{array}{ccc} & & -E_{I_{1}^{T}∼P_{D1}\left(truth\right)}\log\left(D_{\theta_{D1}}\left(I_{1}^{T}\right)\right)\\ & & -E_{I_{1}^{S}∼P_{G1}\left(fake\right)}\log\left(1-D_{\theta_{D1}}\left(G_{\theta_{G1}}\left(I_{1}^{S}\right)\right)\right) \end{array}$$

(6) $$\begin{array}{ccc} & & -E_{I_{2}^{T}∼P_{D2}\left(truth\right)}\log\left(D_{\theta_{D2}}\left(I_{2}^{T}\right)\right)\\ & & -E_{I_{2}^{S}∼P_{G2}\left(fake\right)}\log\left(1-D_{\theta_{D2}}\left(G_{\theta_{G2}}\left(I_{2}^{S}\right)\right)\right) \end{array}$$

#### 2.4.2 Training with attention blocks

Through the backbone segmentor selection scheme, DAU‐Net is selected as the backbone architecture in our proposed algorithm. To further improve segmentation performance, several attention blocks are integrated into the DAU‐Net. Specifically, one, two, and three attention blocks are placed after the last, the last two, and the last three deconvolutional layers in the segmentation model. The corresponding structures are respectively called ADAU‐Net‐A1, ADAU‐Net‐A2, and ADAU‐Net‐A3, and they are shown in the second stage of Figure 1. The framework of the integrated attention blocks is displayed in Figure 1. The input features *I*∈*C*×*H*×*W* is first reshaped into *I′*∈*C*×*N* using a 1×1 convolutional kernel, where *N = H*×*W*. Then, *I′* is transposed into *I′′*∈*N*×*C*, and a matrix multiplication of *I′* and *I′′* is conducted. Next, this output is processed by a SoftMax function to obtain an attention map $A_{mn}$, as defined in Equation (7).

(7) $$\left(e^{I_{n}\times I_{m}}\right)/\left(\sum_{n\;=\;1}^{N}e^{I_{n}\times I_{m}}\right)$$

Subsequently, matrix multiplication of the transpose of $A_{mn}$ and *I′* is performed, and then this output is reshaped into *C*×*H*×*W* named ${A_{mn}}^{\prime }$. Finally, *I* is added to a *δ* multiple of ${A_{mn}}^{\prime }$ in an element‐wise manner to obtain the final result, as defined in Equation (8).

(8) $$\delta \times \sum_{n\;=\;1}^{N}\left(A_{mn}^{\prime }I_{n}\right)+I_{m}$$

The integration of the attention mechanism enhances the interdependency among the pixels in images scans, which highlights the dominant parts and improves the potent information representation for segmentation. Among the three architectures mentioned above, ADAU‐Net‐A2 achieves the most satisfactory results, and it is selected as the final version to be used in our proposed algorithm.

## 3 EXPERIMENTS AND RESULTS

### 3.1 Ablation studies

#### 3.1.1 Backbone segmentor selection scheme

**FIGURE 3:**
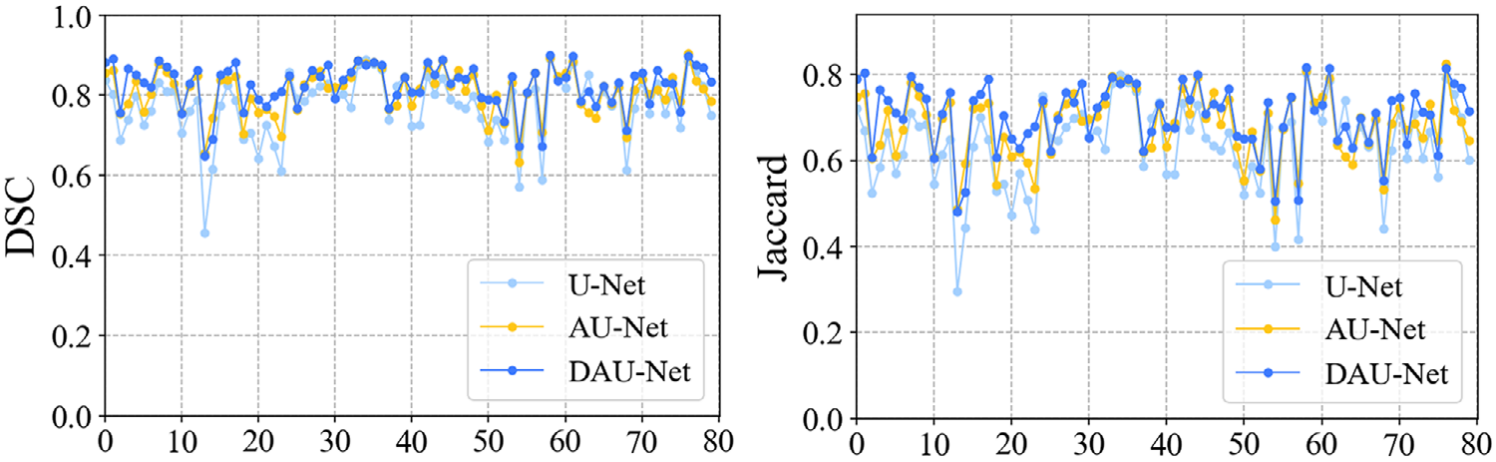
Curves of indexes dice similarity coefficient (DSC) and Jaccard for models U‐Net, adversarial U‐Net (AU‐Net), and duplex adversarial U‐Net (DAU‐Net), respectively. The X‐axis represents the total cases in the testing process

**FIGURE 4:**
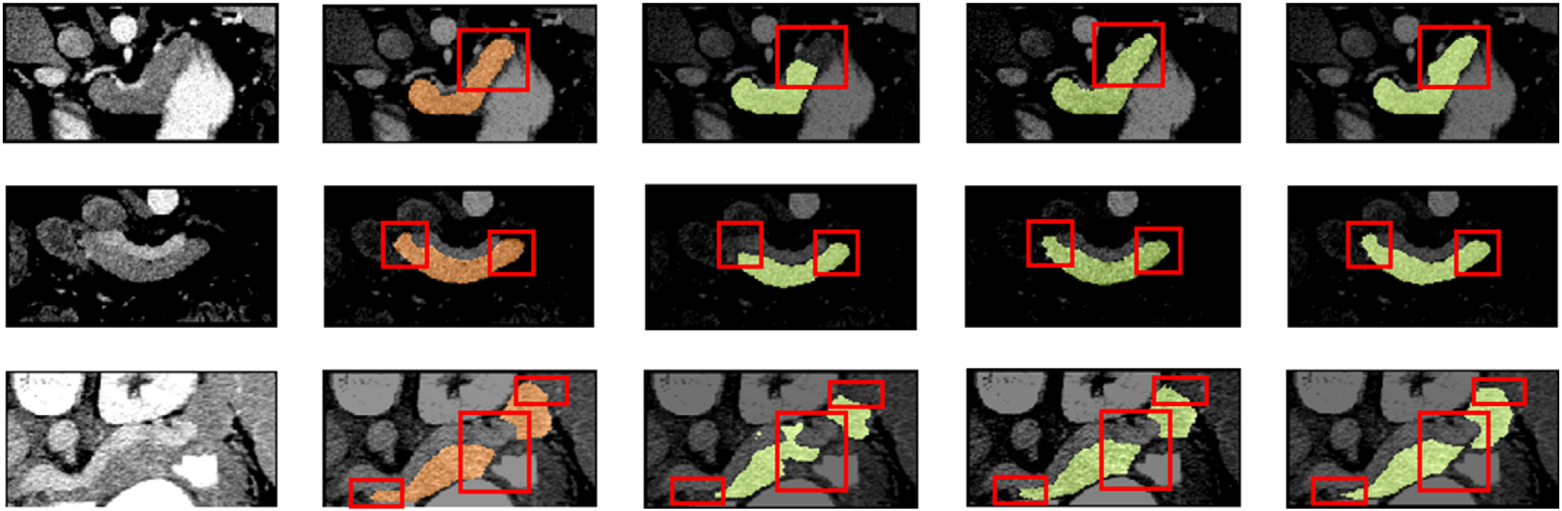
The two‐dimensional (2D) visualization segmentation results comparisons of models U‐Net, adversarial U‐Net (AU‐Net), and duplex adversarial U‐Net (DAU‐Net) from the National Institutes of Health (NIH) dataset #Case56_Slice13, # Case41_Slice32, and # Case73_Slice19 (from top to down). The first and the second columns are original images from the NIH pancreas dataset and the corresponding ground truths. The third, fourth, and fifth columns are segmentation results from models U‐Net, AU‐Net, and DAU‐Net, respectively

In this section, three groups of models are established for pancreas segmentation, namely U‐Net, AU‐Net, and DAU‐Net. To select the best segmentor, several experiments are conducted on the three frameworks. The U‐Net, AU‐Net, and DAU‐Net respectively achieve mean DSC values of 77.37%, 80.83%, and 82.38%, and mean Jaccard values of 63.73%, 68.18%, and 70.39%. Figure 3 illustrates the curves of the DSC and Jaccard coefficient for the models. Figure 4 shows the 2D visualization results of these models for an intuitive observation of the segmentation performance.

#### 3.1.2 Training with attention blocks

**FIGURE 5:**
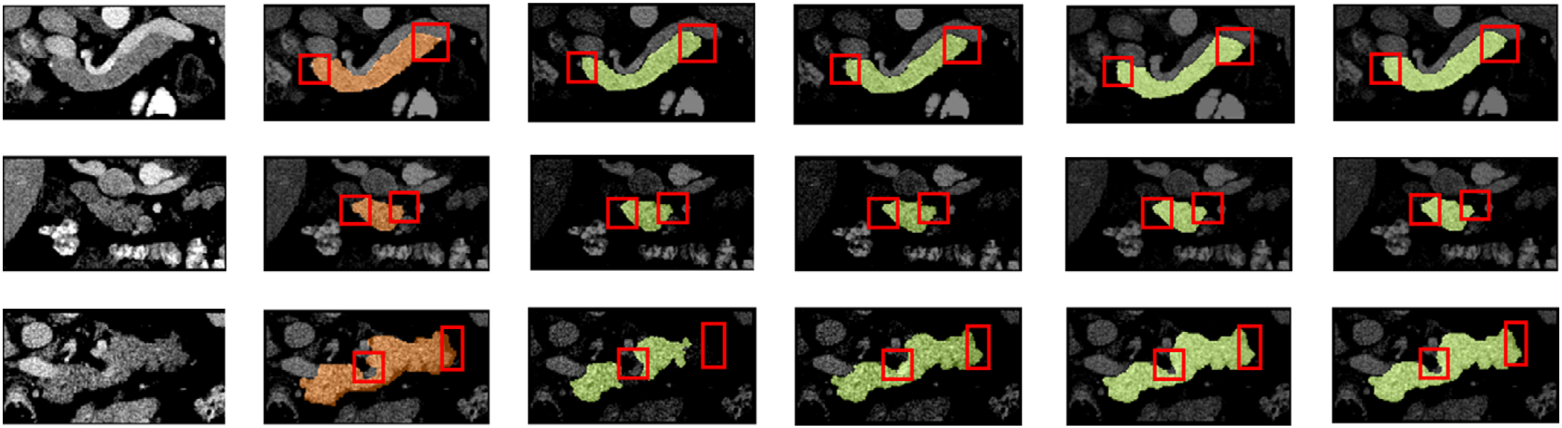
The two‐dimensional (2D) visualization segmentation results comparisons of models U‐Net, adversarial U‐Net (AU‐Net), duplex adversarial U‐Net (DAU‐Net), and attention‐guided duplex adversarial U‐Net (ADAU‐Net) from NIH dataset #Case75_Slice15, #Case49_Slice 61, and #Case69_Slice18 (from top to down). The first and the second columns are original images from the National Institutes of Health (NIH) pancreas dataset and the corresponding ground truths. The third, fourth, fifth, and sixth columns are segmentation results from models U‐Net, AU‐Net, DAU‐Net, and ADAU‐Net, respectively

Based on the backbone segmentor selection scheme, the selected model is trained with attention blocks to improve its segmentation ability. As for the structures of DAU‐Net, ADAU‐Net‐A1, ADAU‐Net‐A2, and ADAU‐Net‐A3, several tests are designed to evaluate their segmentation performance. The detailed numerical values of DSC, Jaccard, ASD, and RMSE for these four models are respectively listed in Table 1, which include the mean, minimum, maximum, and standard deviation values for these indexes in each model. The mean DSC values of DAU‐Net, ADAU‐Net‐A1, ADAU‐Net‐A2, and ADAU‐Net‐A3 are respectively 82.38%, 82.68%, 83.76%, and 82.96%, and their corresponding mean Jaccard values are 70.39%, 70.84%, 72.38%, and 71.27%. The mean ASDs of DAU‐Net, ADAU‐Net‐A1, ADAU‐Net‐A2, and ADAU‐Net‐A3 are 1.22, 1.09, 1.07, and 1.19 mm, and their corresponding mean RMSEs are 2.26, 2.21, 2.17, and 2.23 mm. Figure 5 shows the 2D visualization results of the segmentation models mentioned above. To verify the effectiveness of our proposed model, ADAU‐Net is compared with the‐state‐of‐art methods for pancreas segmentation, and the comparison results are listed in Table 2.

**TABLE 1:** Evaluation results on dice similarity coefficient (%), Jaccard (%), average symmetric surface distance (mm), and Root‐Mean‐Squared Error (mm) of different segmentation models on the National Institutes of Health (NIH) pancreas dataset

**Models**	**DSC**	**Jaccard**	**ASD**	**RMSE**
DAU‐Net	82.38 ± 5.46[70.01, 89.39]	70.39 ± 7.58[54.05, 80.82]	1.22 ± 0.32[0.58, 2.71]	2.26 ± 0.35[1.45, 3.65]
ADAU‐Net‐A1	82.68 ± 5.55[69.54, 89.94]	70.84 ± 7.65[53.71, 81.74]	1.09 ± 0.18[0.55, 2.20]	2.21 ± 0.34[1.44, 3.58]
ADAU‐Net‐A2	83.76 ± 4.94[71.60, 90.25]	72.38 ± 6.95[56.08, 82.24]	1.07 ± 0.23[0.49, 2.31]	2.17 ± 0.32[1.38, 3.52]
ADAU‐Net‐A3	82.96 ± 5.51[68.49, 89.85]	71.27 ± 7.58[52.28, 81.61]	1.19 ± 0.29[0.53, 2.65]	2.23 ± 0.38[1.43, 3.76]

Abbreviations: ADAU‐Net, attention‐guided duplex adversarial U‐Net; ASD, average symmetric surface distance; AU‐Net, adversarial U‐Net; DAU‐Net, duplex adversarial U‐Net; DSC, dice similarity coefficient.

**TABLE 2:** Evaluation results comparisons on dice similarity coefficient (%) of different segmentation methods on the National Institutes of Health (NIH) pancreas dataset

**DSC**	**[21]**	**[5]**	**[7]**	**[6]**	**[24]**	**[23]**	**[22]**	**[25]**	**Ours**
Mean	71.8	78.01	83.18	83.70	85.32	84.50	83.06	85.46	**83.76**
Std	10.7	8.20	4.81	5.10	4.19	4.97	5.57	4.80	**4.94**
Min	25.0	34.11	65.10	59.00	71.04	62.81	67.96	67.03	**71.60**
Max	86.9	88.65	91.03	91.00	91.47	91.02	90.37	92.24	**90.25**

Abbreviation: DSC, dice similarity coefficient.

## 4 DISCUSSION

### 4.1 Backbone segmentor selection scheme

It is challenging to segment organs from biomedical imaging, especially for the pancreas that has a small volume and a large variation in shape. To achieve better segmentation results, a backbone segmentor selection scheme is firstly designed to select an effective backbone framework for pancreas segmentation from the models of U‐Net, AU‐Net, and DAU‐Net. The obtained numerical result indicates that DAU‐Net performs better for pancreas segmentation than U‐Net and AU‐Net. Figure 3 shows the value distributions curves of the DSC and Jaccard coefficient for these three models. It can be seen that DAU‐Net achieves a higher mean level of DSC and Jaccard coefficient than U‐Net and AU‐Net. Figure 4, especially the red highlighted areas, shows that the prediction maps obtained from U‐Net lose chunks of information for organs, while the DAU‐Net selected through the backbone segmentor selection scheme can capture more details to make up for the information loss in U‐Net and AU‐Net, thus effectively improve the overall outline of organs. The improvement of the segmentation results shown in Figure 4 indicates that the integration of DAU‐Net effectively improves the segmentation performance compared to U‐Net and AU‐Net. This confirms the significance of the backbone segmentor selection scheme and the contributions of DAU‐Net on pancreas segmentation.

### 4.2 Training with attention blocks

After the selection of the backbone segmentor, the attention mechanism is integrated into DAU‐Net to improve the network performance. From the numerical values, it is obvious that our proposed DAU‐Net, ADAU‐Net‐A1, ADAU‐Net‐A2, and ADAU‐Net‐A3 severally exceed the basic segmentation model U‐Net with DSC values of 5.01%, 5.31%, 6.39%, and 5.59%, which demonstrates that these proposed four models are effective for improving pancreas segmentation performance. It can be seen from Table 1 that the mean DSC value of ADAU‐Net‐A2 outperforms DAU‐Net, ADAU‐NetA1, and ADAU‐Net‐A3 by 1.38%, 1.08%, and 0.8%. Also, the mean Jaccard value of ADAU‐Net‐A2 is 1.99%, 1.54%, and 1.11% higher than that of the other three groups. This indicates that compared with the other three models, the prediction maps obtained from ADAU‐Net‐A2 have a higher similarity with their corresponding ground truths. Their mean ASD and mean RMSE values indicate that compared with the other three models, the pancreas edge in the prediction maps obtained from ADAU‐Net‐A2 is better segmented referring to the edge of the ground truths. ADAU‐Net‐A2 achieves the optimal results among the four models. This is because the integration of two attention blocks in ADAU‐Net‐A2 helps highlight the most informative features and make the prediction contextualized, thus improving the segmentation performance. ADAU‐Net‐A3 further selects features based on the results of ADAU‐Net‐A2. However, it results in the reduction of the useful information that is greatly fitted for pancreas segmentation in ADAU‐Net‐A2. Figure 5, especially the red highlighted areas, shows that the prediction maps obtained from DAU‐Net are still deficient in the wispy positions, while the selected ADAU‐Net trained with attention blocks can collect more details to smooth the organ outlines to resemble the ground truths. The distinct refinement of the segmentation results shown in Figure 5 demonstrates the contributions of the attention blocks for pancreas segmentation.

The improvements among these four different groups of models in “training with attention blocks” are much less pronounced than models in the “backbone segmentor selection scheme”. This is because there is a lot of room for improvement on the most basic segmentation model U‐Net, thus the optimal model DAU‐Net in “backbone segmentor selection scheme” achieves a DSC score of 82.38%, which is 5.01% higher than the basic U‐Net. As it is recognized as a challenging task to further improve AI‐based approaches in medical imaging processing for pancreas segmentation when the best DSC recorder is above 0.8 or higher. Therefore, on the basis of the DAU‐Net, the room for improvement is relatively limited. Despite the difficulty, ADAU‐Net‐A2 still improves the segmentation performance and achieves a DSC score of 83.76%, which effectively indicates that our proposed attention‐guided duplex adversarial U‐Net is a potential tool for pancreas segmentation.

The proposed ADAU‐Net is compared with the‐state‐of‐art models for pancreas segmentation to evaluate its segmentation performance, and the comparison is conducted on the NIH datasets. Table 2 lists the numerical values of the relevant models, and it can be observed that ADAU‐Net achieves an optimal DSC value of 83.76%. The holistically‐nested CNN proposed by Roth et al.^5^ achieves a DSC value of 78.01%, which is 5.75% lower than that of our method. The CNN‐RNN architecture proposed by Cai et al.^6^ obtains an optimal DSC with a mean value of 83.70% and a standard deviation value of 5.10%, while ADAU‐Net achieves a mean DSC value of 83.76% and a standard deviation value of 4.94%. The result indicates that our method performs better and is more stable. The DSC value of the multi‐level deep convolutional network proposed by Roth et al.^22^ is 11.96% lower than that of our algorithm. ADAU‐Net improves the DSC score of 83.06% obtained from an adversarial model under two‐tier constraints^23^ to a DSC of 83.76%. The above comparisons sufficiently indicate that the proposed ADAU‐Net is a satisfactory and promising model for pancreas segmentation. The recurrent saliency transformation network proposed by Yu et al.^24^ achieves a higher mean DSC but lower standard deviation than ADAU‐Net, indicating that our proposed model has a much stable segmentation performance.

Although our proposed model shows competitive performance compared with most pancreas segmentation methods, it still needs to be further improved. As can be seen from Table 2 that the novel Bayesian model proposed by Ma et al.^25^ and the globally guided progressive fusion network proposed by Fang et al.^26^ outperform ADAU‐Net in DSC score by 1.56% and 1.7%. Future research will investigate the effect of the attention blocks with different inner structures on our segmentation models and attempt to improve our existing network by exploiting novel methods. Besides, future research will attempt to build our model on a 3D network to explore its potential for pancreas segmentation.

## 5 CONCLUSION

To our best knowledge, this paper is the first to present an attention‐guided dual adversarial network for pancreas segmentation. To implement this algorithm, a backbone segmentor selection scheme is first introduced to select an optimal backbone segmentor from the three segmentation model variants based on the conventional U‐Net. Then, several groups of attention blocks are integrated into the selected backbone segmentor at different positions to collect as much contextual information as possible. The proposed ADAU‐Net is trained on the pubic NIH Pancreas‐CT dataset under 4‐fold cross‐validation. The experiments results show that ADAU‐Net outperforms all the segmentors considered in this work in terms of DSC, Jaccard coefficient, ASD, and RMSE. Also, it achieves competitive performance compared to the‐state‐of‐art architectures for pancreas segmentation.

## CONFLICT OF INTEREST

The authors declare that they have no conflict of interest.

## AUTHOR CONTRIBUTIONS

Meiyu Li designed the network architecture, performed the algorithm, and wrote the manuscript. Fenghui Lian, Yang Li, and Shuxu Guo were involved in analyzing experimental results and reviewing the work. All authors have approved the submitted manuscript.

## Supporting information

Supporting Information

## Data Availability

The data that support the findings of this study are openly available in Cancer Imaging Archive ‐ Pancreas‐CT at https://doi.org/10.7937/K9/TCIA.2016.tNB1kqBU.^18^
